# Aminated reduced graphene oxide-CuFe_2_O_4_ nanohybride adsorbent for efficient removal of imidacloprid pesticide

**DOI:** 10.1039/d4ra03720k

**Published:** 2024-10-07

**Authors:** Hisham S. M. Abd-Rabboh, Ayman H. Kamel

**Affiliations:** a Chemistry Department, College of Science, King Khalid University Abha 62223 Saudi Arabia; b Chemistry Department, Faculty of Science, Ain Shams University PO Box 11655 Cairo Egypt ahkamel76@sci.asu.edu.eg +201000361328; c Chemistry Department, College of Science, University of Bahrain Sakhir 32038 Bahrain ahmohamed@uob.edu.bh +97332085874

## Abstract

To remove organic and inorganic agrochemicals from contaminated soil and water, adsorption has been regarded as a viable remediation approach. For the removal of organic pollutants, such as pesticides, cost-effective adsorbents have garnered a lot of interest. These include waste-derived materials, clay composites, metal–organic frameworks (MOFs), nanocomposites, and biochar-modified materials. In this study, copper ferrite (CuFe_2_O_4_) was prepared, characterized, and modified with aminated reduced graphene oxide (Am-rGO) to form a CuFe_2_O_4_/Am-rGO nanocomposite for the effective removal of imidacloprid (IMD) from water. The Langmuir isotherm model was used to determine the maximum adsorption capacity of the adsorbent (CuFe_2_O_4_/Am-rGO), which was estimated to be 13.1 (±1.5) mg g^−1^. At 0.5 mg L^−1^ IMD, the adsorbents were able to extract up to 97.8% of the IMD from the aqueous solution. The Freundlich model and the pseudo second-order model agreed well with the experimental data, proving that physisorption and chemosorption both played a role in the sorption process. CuFe_2_O_4_/Am-rGO nanocomposite offers high stability and improved reusability due to its improved removal efficiency. After five adsorption–desorption cycles, there was no appreciable reduction in elimination. Additionally, after adsorption tests, IMD can be easily removed after adsorption by an external magnetic field. These showed that Am-rGO had changed the surface of CuFe_2_O_4_ to make it easier for IMD to stick to it in aqueous solutions. When used adsorbent is co-processed with ethanol extraction and ultrasound cavitation, it can be regenerated and still work well as an adsorbent. Furthermore, CuFe_2_O_4_/Am-rGO demonstrated its environmental safety and ability to continue absorbing IMD across a variety of diverse matrices. As a result, this study demonstrates that CuFe_2_O_4_/Am-rGO is a long-lasting, easily prepared, and efficient adsorbent for the removal of IMD as one of the neonicotinoids.

## Abstract 2

To remove organic and inorganic agrochemicals from contaminated soil and water, adsorption has been regarded as a viable remediation approach.

## Graphical abstract

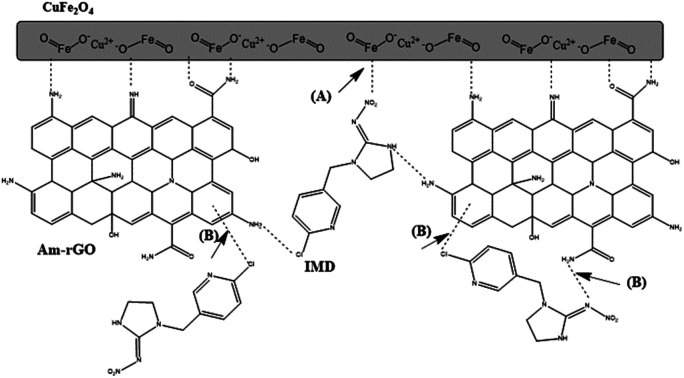

## Introduction

Imidacloprid (IMD), the first neonicotinoid insecticide, was originally registered as a commercial product in 1990.^1,2^ It has been extensively used on soils, plants, and seeds. Nonetheless, a great deal of information has been published about how IMD affects non-target species and the ecosystem.^3^ Over the past 20 years, there has been a significant increase in the detection of high residues of IMD at varying concentrations in several matrices, including soil.^4^ The degrading half-life of IMD in soil varies from 40 to 1230 days, depending on the soil content and characteristics.^5^ In addition, the ensuing hazards to both terrestrial and aquatic invertebrates are also cause for concern.^6^ IMD can have negative effects on aquatic and terrestrial creatures, according to several studies.^7^ Previous research found that IMD was very harmful to earthworms, with an LC50 value of 2.26 mg kg^−1^ after 14 days and an AC50 value of 1.34 mg kg^−1^ after 2 days. This is the 50% effect concentration of avoidance rate.^8^ Additionally, Zhang *et al.* (2014) and Wang *et al.* (2016)^3,9^ discovered that sub-chronic exposure to IMD can cause lipid peroxidation, DNA damage, and oxidative stress in earthworms. Additionally, due to imidacloprid's systemic nature, its metabolites can be distributed throughout the plant, and its presence has been found in some plants' nectar and pollen.^10–13^ Recent observations have shown that the widespread usage of IMD reduced honey bee colony health and immunity.^14^ Additionally, due to the widespread usage of IMD, concentrations of up to 320 μg L^−1^ have been found in surface waters all over the world (including Europe and America).^15,16^ IMD is currently regarded as an emerging contaminant (EC) and is listed in the second section of the European Surface Water Watch List because of its high toxicity, solubility, and persistence.^17^ IMD concentrations in surface water ranged from ng L^−1^ to μg L^−1^,^16^ whereas earlier studies on IMD adsorption onto other adsorbents (such as corn cob and bamboo chips, peanut shell, and amino functionalized silica nano hollow sphere) were primarily focused on high concentrations (1–60 mg L^−1^) of IMD removal.^15,18,19^ This was a result of the analytical method's limitations or its exclusion from most countries' regulations.^18^

The majority of emergent contaminants (ECs) in wastewater were discharged into the aquatic ecosystem since conventional treatment technologies did not have the capacity to remove them, which raised the potential ecological negative impacts on the environment.^20^ Adsorption,^21,22^ advanced oxidation,^23,24^ and biodegradation^25^ were all used to get rid of ECs. Adsorption was one of them, and it was thought to be a trustworthy method because of its inherent benefits of low cost, high efficiency, and ease of use.^26,27^ In addition, adsorption could also prevent the emergence of harmful degradation products, which could result in environmental pollution.^28^ The use of nanomaterials to remove environmental contaminants is currently an area of interest. Based on their distinguishing features, particularly their large surface area, high adsorption, and distinct photoelectric property. However, because of their small particle size, they are difficult to separate from aqueous solutions, which limit their use in water treatment. Utilizing magnetic nanoparticles that can be easily isolated from solutions using an external magnetic field is therefore favored.^29^ Modifications can significantly improve the surface characteristics of nanoparticles. This is favored because the modified material and the reduced solvent shielding of the ions in the interlamellar environment have a van der Waals interaction.^30^ Chitosan, carbon nanotubes, and graphene oxide (GO) have recently drawn more interest as potential doping materials. Due to its distinctive physical–chemical properties, such as its light weight, high mechanical strength, large surface area, abundance of functional groups (–COOH, –OH, NH_2_, epoxy(–CH(O)CH)), high hydrophilicity, and favorable biocompatibility, GO stands out among the others as a suitable composite material.^31^ After adsorption, modified CuFe_2_O_4_ demonstrated good separation performance.^23,29,32^ According to these studies, CuFe_2_O_4_ modified adsorbent separated from solution efficiently and quickly due to its higher magnetic sensitivity than Fe_3_O_4_ modified adsorbent.

Chemical removal of most oxygen-containing groups in graphene oxide (GO) creates reduced graphene oxide (rGO), a material with increased electrical conductivity.^31^ However, scientists frequently attempt to further modify its characteristics with amine groups for specific purposes. Adding amino groups to rGO makes it more water-friendly and easy to mix with organic and water-based solvents, which is important for processing and making composite materials with rGO. Amine-functionalized rGO offers chemically reactive sites that can be used to connect different functional groups or nanoparticles.^33^ As amine groups help polymer matrices or other materials interact more strongly, they can also improve the mechanical properties of rGO-based composites.^33^ Magnetic nanoparticles, such as iron oxide (Fe_3_O_4_), can functionalize rGO through covalent or non-covalent interactions.^29,32^ These nanoparticles may bind to rGO because of the presence of amine groups on the surface. External magnetic fields can alter the material when rGO and magnetic nanoparticles combine. This is helpful for operations including magnetic separation, targeted medication administration, and environmental cleanup.

In this study, the material CuFe_2_O_4_/Am-rGO nanocomposite were successfully synthesized and characterized by SEM, BET, XRD, and FT-IR, and then the application of the material for the removal of IMD from aqueous solutions was investigated by the influence of different adsorption parameters. The produced adsorbents' removal effectiveness was examined, and their adsorption and desorption behaviors towards IMD species have been investigated.

## Experimental section

### Apparatus

The JEOL-JEM-2100 electron microscope apparatus (Osaka, Japan) was used to capture high-resolution transmission electron microscopy (HRTEM) images. By applying (CuK radiation = 0.154 nm) in the angular area of 2 = 4–80, an X-ray diffractometer of the second generation, the BRUKER D2 PHASER (Berline, Germany), was used to characterize the produced adsorbents. The operating parameters were 40 kV, 40 mA, and an 8 min^−1^ scanning rate. N_2_ adsorption and desorption measurements of the Brunauer–Emmett–Teller (BET) surface area were performed at 77 K using the Nova 3200 s unite instrument and relative pressure (*p*/*p*^o^) at 0.25104.

### Chemicals

All the chemicals were of analytical reagent grade and were used directly without further purification. Sigma-Aldrich (St. Louis, MO, USA) provided the pesticide imidacloprid (PESTANAL®, analytical standard), and Am-rGO.

### Preparation of CuFe_2_O_4_/Am-rGO nanocomposite

The co-precipitation approach was used to produce the nanoparticles.^34^ In 200 mL of a 1% by weight PEG solution, 11.7 mmol of CuSO_4_ and 14.98 mmol of FeCl_3_ were dissolved. To ensure that all the components were in equilibrium, the solution was stirred continuously for nearly an hour. 4 M KOH was added to the mixture drop-by-drop while being vigorously stirred until pH 9 was reached. After two more hours of magnetic stirring, the mixture was aged for the next day. The precipitate was filtered, thoroughly cleaned with distilled water to remove any Cl^−^ and SO_4_^−2^ ions, and then dried for two hours at 70 °C. Following precipitation, the resulting product was calcined at 600 °C in air for three hours before being milled to produce a fine powder.

A CuFe_2_O_4_/Am-rGO nanocomposite was synthesized in detail as follows: a suspension containing 0.25 g of Am-rGO was sonicated in an ultrasound homogenizer (400 W) for 60 min with 12 min pulse intervals. Following the procedure, 5.0 g of CuFe_2_O_4_ was added to the suspension, which was then continuously agitated at a rate of 500 rpm for 60 minutes before being filtered and dried. The dry material was pulverized and sieved (0.15 mm) before being placed into the tube furnace to repeat the pyrolysis process described above (60 min).

### Adsorption experiments

Batch adsorption experiments were carried out to determine the effects of the adsorbent dose, solution pH, and initial IMD concentrations on the rate of IMD removal. The details of the trials, which were conducted in triplicate, are as follows:

(1) Effect of adsorbent amount: *C*_0_ = 0.5 mg L^−1^, *V* = 50 mL, *m* = 0.005–0.05 g, and *t* = 120 min.

(2) Kinetics: *t* = 0–250 min, *C*_0_ = 5.0 mg L^−1^, *V* = 50 mL, and *m* = 0.02 g.

(3) Adsorption isotherms: *C*_0_ = 0.1–10 mg L^−1^, *V* = 50 mL, *m* = 0.02 g, and *t* = 120 min are the isotherms.

The removal rate of IMD (*R*) and adsorbent capacity (*q*_*t*_) were determined using eqn (1) and (2), respectively.

1 % *R* = (*C*_o_ − *C*_*t*_)/*C*_o_ × 100

2 *q*_*t*_ = (*C*_o_ − *C*_*t*_)/*m* × *V*

where *q*_*t*_ (mg g^−1^) was the amount of adsorption at time *t*, and *C*_0_, *C*_*t*_ (mg L^−1^) were the IMD concentrations at start and time *t*, respectively. The solution volume was given as *V* (L), dose of adsorbent was given as *m* (g), and contact time was given as *t* (min). We used 0.01 M NaOH and HCl to change the pH of the solution.

Samples were taken during the batch experiments at the proper intervals. After filtering all the obtained samples *via* a 0.22 μm membrane, high-performance liquid chromatography (HPLC) was used to measure the IMD concentrations. The isocratic mobile phase had a flow rate of 0.6 mL min^−1^ and included 0.1% formic acid in water (v/v, 30%) and acetonitrile (v/v, 70%).

For the reusability study, the mixture containing the adsorbent and IMD is spun at high speeds till the adsorbent settles at the bottom. A magnetic field is applied to separate the adsorbent from the liquid phase quickly. After separation, the adsorbent is washed with either water or ethanol to remove any loosely bound IMD or impurities. The washed adsorbent is then dried in an oven at 60 °C before being reused in the next cycle. For a new adsorption process to be performed after each cycle, a fresh solution containing 0.5 mg L^−1^ (50 mL) of IMD is introduced with 0.02 g of the dried and cleaned adsorbent. The adsorption process is repeated under the same conditions as the initial experiment. This includes maintaining the same temperature, pH, stirring speed, and contact time. After each cycle, the adsorption efficiency is determined by measuring the concentration of IMD in the solution before and after the adsorption process using the abovementioned HPLC method. The adsorption capacity of the adsorbent is compared across multiple cycles to assess its reusability.

## Results and discussion

### Characterization of the adsorbent

#### X-ray diffraction (XRD) pattern

**Fig. 1:**
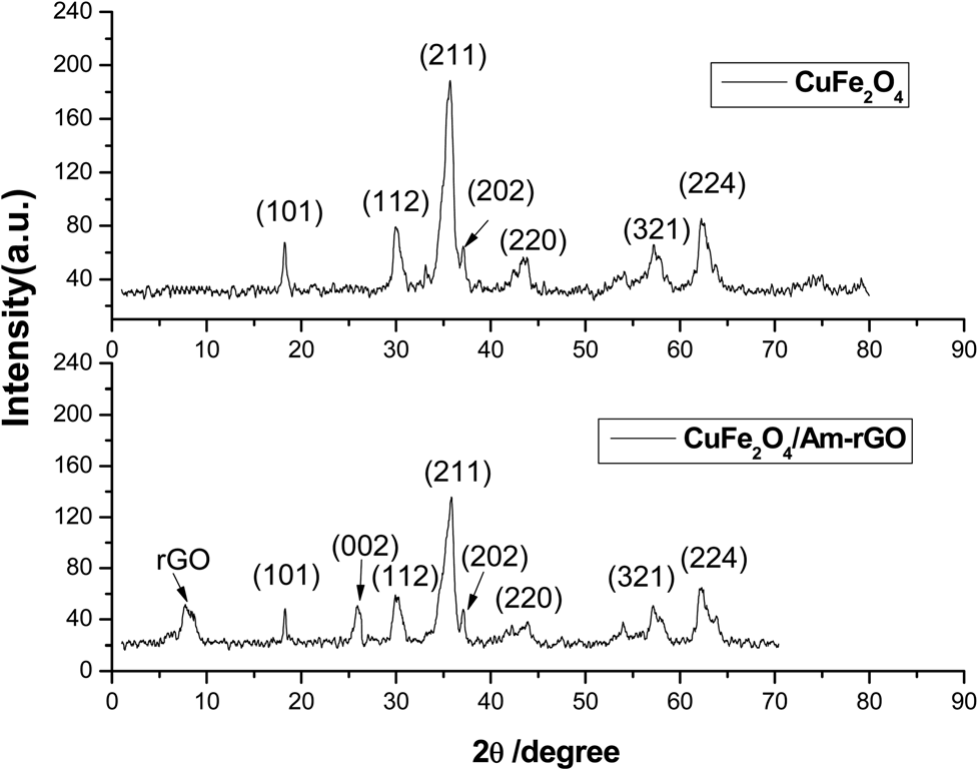
XRD pattern of both CuFe_2_O_4_ and CuFe_2_O_4_/Am-rGO nanocomposite.

XRD is used to figure out the crystalline phase of CuFe_2_O_4_ and CuFe_2_O_4_/Am-rGO nanocomposites. The results are shown in Fig. 1. The XRD pattern of CuFe_2_O_4_ showed values at 18.36, 29.98, 34.69, 35.66, 37.21, 43.84, 58.14, and 62.33 that indexed to the planes of a face-centered cubic CuFe_2_O_4_ (JCPDS No. 34-0425) at (1 0 1), (1 1 2), (2 1 1), (2 0 2), (2 2 0), (3 2 1), and (2 2 4), respectively. The XRD pattern of CuFe_2_O_4_/Am-rGO shows a sharp diffraction peak at 26.4 with a *d*-spacing of 3.35 Å, which represents the characteristic (002) plane peak of hexagonal graphite (JCPDS number 75-1621), and a peak at 9.87 with a *d*-spacing of 8.79 Å, which indicates that the surface is functionalized with NH_2_ groups.

#### SEM and EDAX analysis

**Fig. 2:**
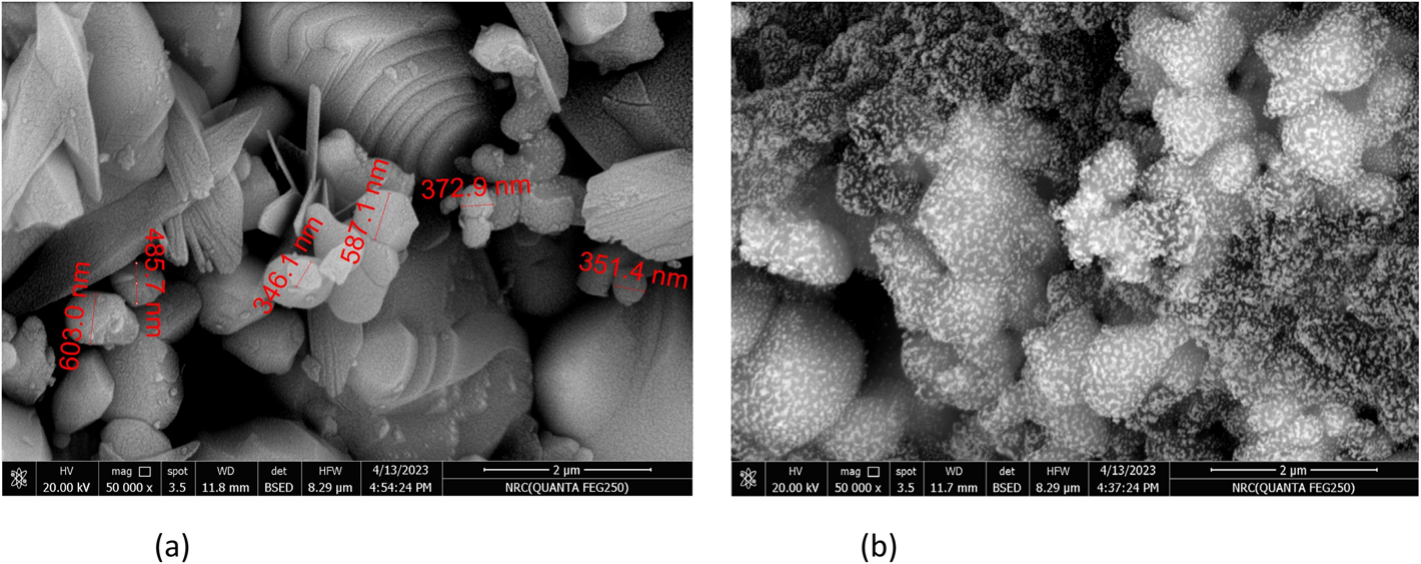
SEM images of (a) CuFe_2_O_4_ and (b) CuFe_2_O_4_/Am-rGO nanocomposite.

The produced CuFe_2_O_4_ and CuFe_2_O_4_/Am-rGO nanocomposite morphologies were characterized by SEM to comprehend the structure–performance relationship. SEM images showing the shape of as-prepared adsorbents are shown in Fig. 2. This synthetic CuFe_2_O_4_ (Fig. 2A) has a considerably different morphology from CuFe_2_O_4_/Am-rGO (Fig. 2B). The latter was made up of particles that ranged in size from 24 to 65 nm, whereas the former had a clearly porous structure.

**Fig. 3:**
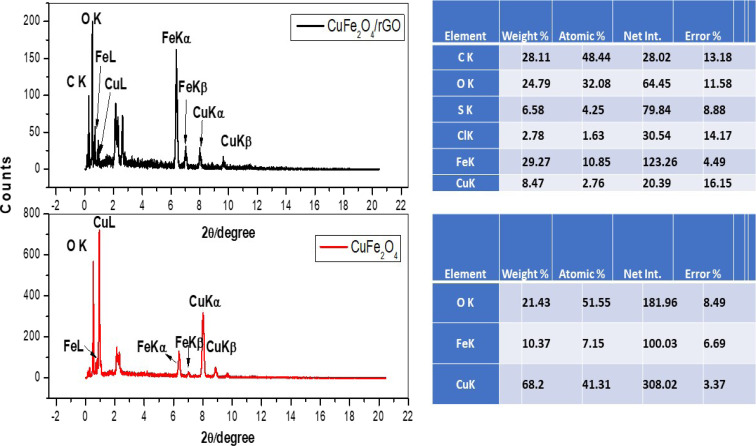
EDAX analyses with the composition of the elements.

The elemental composition of CuFe_2_O_4_ and CuFe_2_O_4_/Am-rGO nanocomposite is also significantly revealed by the EDAX analysis (Fig. 3).

#### Textural properties

**Fig. 4:**
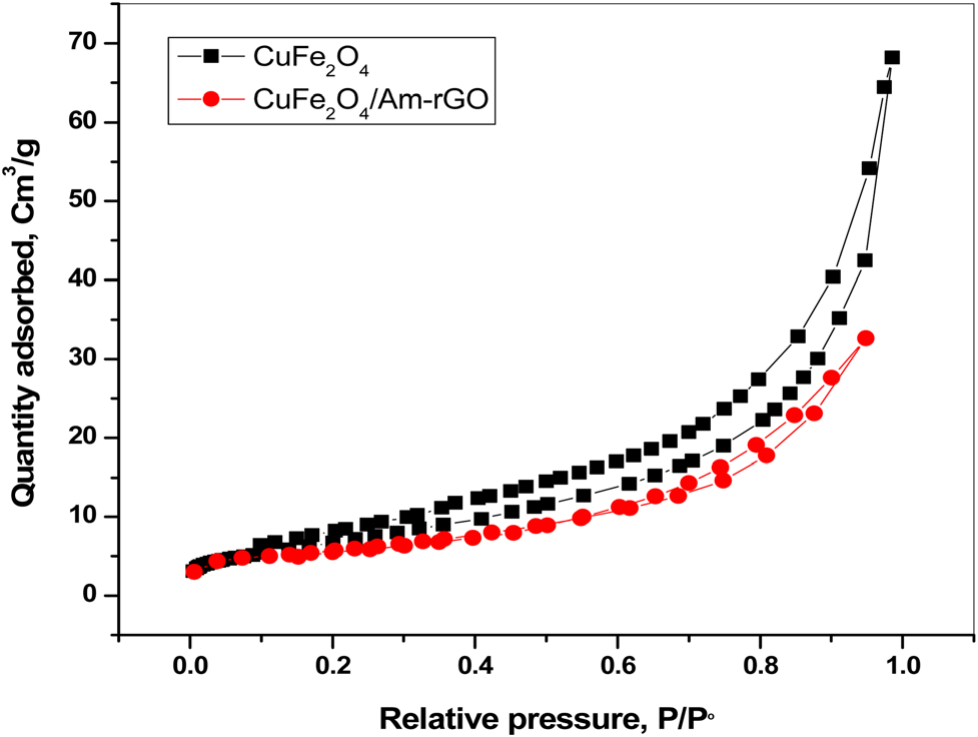
N_2_ adsorption–desorption isotherms of CuFe_2_O_4_ and CuFe_2_O_4_/Am-rGO nanocomposite.

Nitrogen sorption analysis was used to look at the textures of both CuFe_2_O_4_ and CuFe_2_O_4_/Am-rGO nanocomposite. Fig. 4 displays the pore size distribution as well as the nitrogen adsorption–desorption isotherms. The isotherms demonstrate that CuFe_2_O_4_ and CuFe_2_O_4_/Am-rGO nanocomposite both produce type IV isotherms and type H3 hysteresis loops, with the hysteresis shaping more like that for the slit pores and covering a broad relative pressure range, starting from about 0.6 and extending almost to 0.95, indicating their large porosity. The BET surface area of the CuFe_2_O_4_, CuFe_2_O_4_/Am-rGO nanocomposite and Am-rGO is 23.3 ± 1.5, 48.8 ± 1.8 and 54.4 ± 1.3 m^2^ g^−1^, respectively. In addition, the average pore volume is 0.22 ± 0.03, 0.21 ± 0.07 and 0.3 ± 0.02 cm^3^ g^−1^, respectively. This finding implies that creating mesoporous structures using the nano casting technique greatly increases the BET surface areas. CuFe_2_O_4_, CuFe_2_O_4_/Am-rGO nanocomposite and Am-rGO have typical pore diameters of 17.2 ± 0.3, 5.8 ± 0.4 and 11.1 ± 1.2 nm, respectively.

### The pH effect

**Fig. 5:**
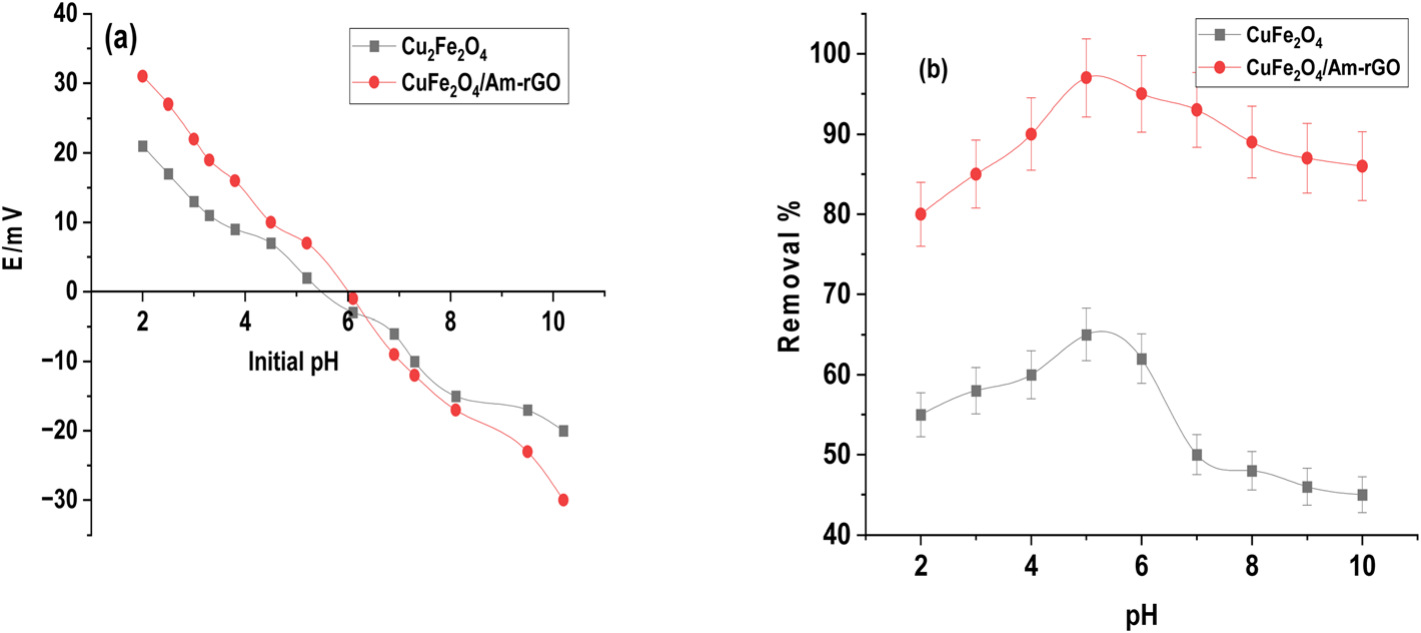
The pH point of zero charge of the synthesized CuFe_2_O_4_ nanoparticles and CuFe_2_O_4_/Am-rGO nanocomposite (a), and the effect of pH on IMD removal rate (b).

We used the pH drift method shown in Fig. 5a to find the pH point of zero charge (pHpzc) of the CuFe_2_O_4_ nanoparticles and CuFe_2_O_4_/Am-rGO nanocomposite that were synthesized. The pH at which the nanoparticles' surface has zero net charges is known as pHpzc. The material's surface would typically be positive and have a strong affinity for negatively charged or anion pollutant species at pH values lower than the pHpzc of the nanoparticles. However, the surface turns negative at pH values greater than pHpzc, which draws cationic or positively charged pollutants. The pHpzc of the CuFe_2_O_4_/Am-rGO nanocomposite and the produced CuFe_2_O_4_ NPs were 6.1 and 5.9, respectively. This means that the surface of the nanoparticles/nanocomposites would be positive at acidic pH values up to pH 6.0, at which point it would turn negative.

IMD is an amphiphilic molecule, with p*K*_a_ values between 1.56 and 11.12.^35^ The IMI^+^ species distribution showed that at pH values less than 1.56, IMD^±^ was the dominant species, followed by IMD^−^ at pH values greater than 11.12. Furthermore, the IMD removal rate increased with pH up to 5.3 and subsequently declined at higher pH values, according to the influence of solution pH (from 2 to 12) shown in Fig. 5b. When the surface charge is close to zero, there is minimal electrostatic repulsion or attraction between the adsorbent and the adsorbate. This can lead to optimal adsorption conditions because the adsorbate can interact with the adsorbent surface more uniformly, without strong repulsive forces pushing it away or strong attractive forces causing it to clump. In addition, the adsorbent surface is more available for adsorption since it doesn't strongly attract or repel the adsorbate. This often results in better distribution of the adsorbate across the adsorbent's surface, leading to higher adsorption capacity and efficiency. So, at pH 5.3, the adsorbents were most effective because their surface charge was nearly neutral, reducing unwanted interactions between the adsorbents and the adsorbate (IMD). This neutral charge allowed the adsorbate to interact with the adsorbent surface more evenly, improving the adsorption process. A similar behavior was seen in the adsorption of IMD onto a silver@graphene oxide nanocomposite; similarly, the amount of IMD adsorbed increased until pH 6.6 and then reduced on both sides.^36^ CuFe_2_O_4_/Am-rGO nanocomposite's adsorption capability at various pH values demonstrated that physicochemical interaction was the primary mechanism in IMD adsorption.

### Effect of adsorbent dosage

**Fig. 6:**
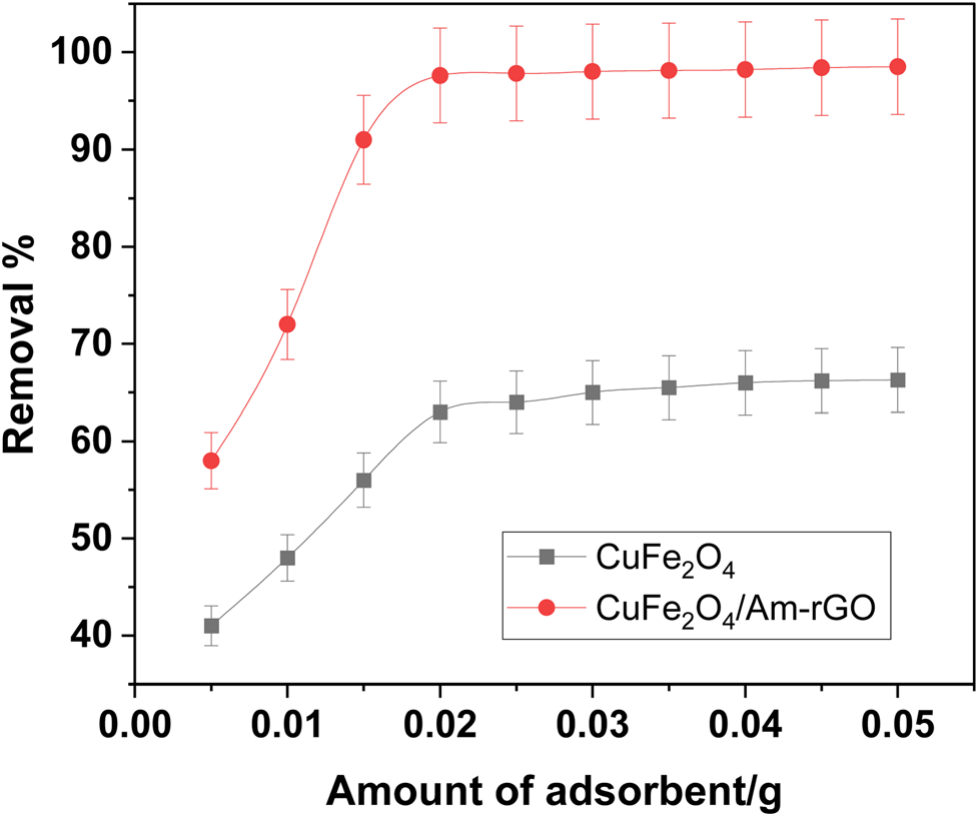
Effect of adsorbent dose on IMD removal from aqueous solution [conditions: initial conc. of IMD solution = 0.5 mg L^−1^, volume = 50 mL, contact time = 120 min, pH = 5.3].

The effect of the adsorbent dose (*m* = 0.005–0.05 g) on the rate of IMD removal are shown in Fig. 6. It was found that the adsorbent dose and contact duration increased the rate of IMD removal. This was because a higher dose made more binding sites available.^29,37^ In the dosage range of 5.0–50 mg, the removal rates of IMD by CuFe_2_O_4_ and CuFe_2_O_4_/Am-rGO were 40.6–66.3% and 57.9.3–99.7% at 120 minutes, respectively. Compared to CuFe_2_O_4_, CuFe_2_O_4_/Am-rGO had a greater adsorption capacity and efficiency. After 120 minutes, the IMD clearance rate with the 0.02 g dose increased to 97.7%. Here, the proper dose of 0.02 g was employed in the kinetic and isotherm investigations for cost-effectiveness.

### Impact of the IMD's initial concentration

**Fig. 7:**
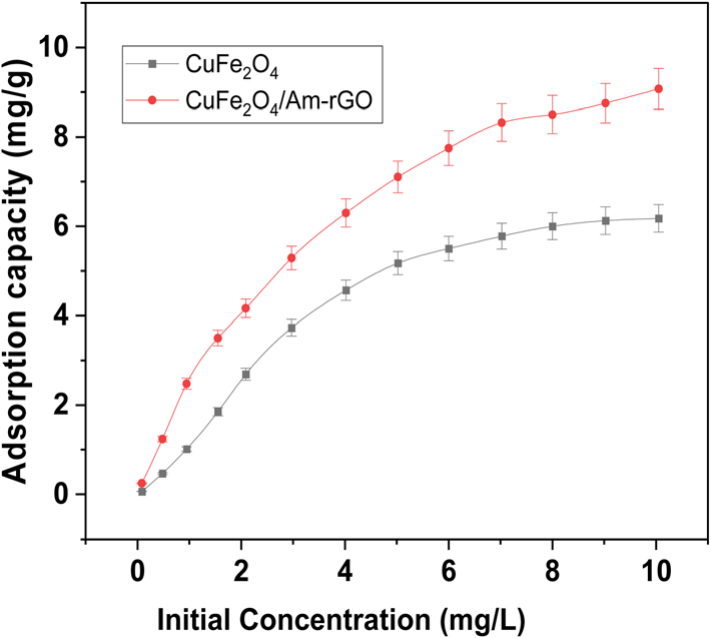
Effect of initial concentration of IMD solution on the adsorption capacity of IMD [conditions: adsorbent dose = 0.02 g, pH = 5.3, volume = 50 mL, contact time = 120].

In both adsorbent types, the optimal experimental conditions were used to examine the impact of IMD concentration at pH 5.3. For duration of 120 min, 50 mL aliquots of IMD solution at pH 5.3 were subjected to an adsorbent dosage of 0.02 grams. Fig. 7 illustrates how the adsorption capacity (*Q*_*t*_) of both adsorbents grew progressively when the IMD concentration was raised, until all each adsorbent's active sites were taken up and no more could be taken up. Both the CuFe_2_O_4_ and the CuFe_2_O_4_/Am-rGO composite had maximum capacities per unit mass (*Q*_max_) of 6.15 ± 0.3 and 9.04 ± 0.2 mg g^−1^, respectively. This demonstrates that the CuFe_2_O_4_/Am-rGO composite is a better option for removing IMD from an aqueous solution than CuFe_2_O_4_.

### Adsorption kinetics

**Fig. 8:**
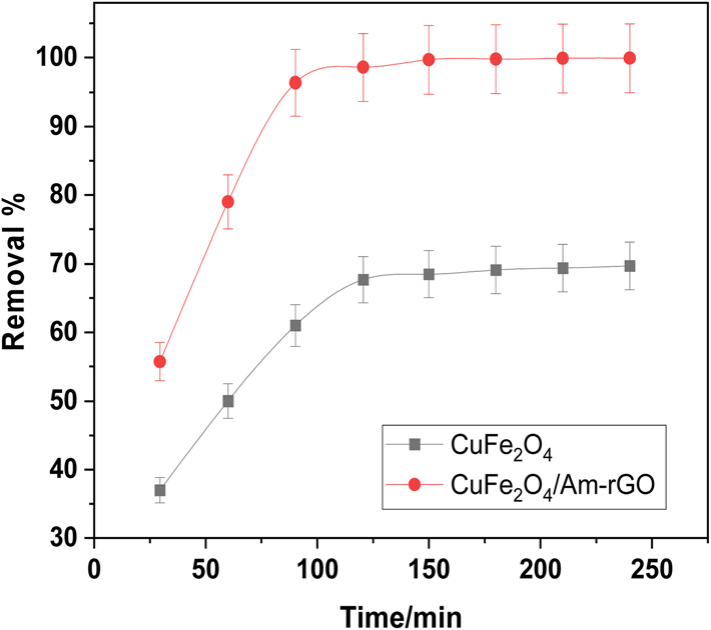
Impact of contact time on the removal of IMD [conditions: initial conc. of IMD solution = 0.5 mg L^−1^, adsorbent dose = 0.02 g, pH = 5.3, volume = 50 mL].

Fig. 8 illustrates how the IMD concentration was examined in relation to each adsorbent's contact time. According to the findings, CuFe_2_O_4_ needed 120 minutes to remove more than 60% of the IMD. On the other hand, 90 minutes were needed to reach equilibrium in the CuFe_2_O_4_/Am-rGO compound, with a 99.7% clearance rate.

**Fig. 9:**
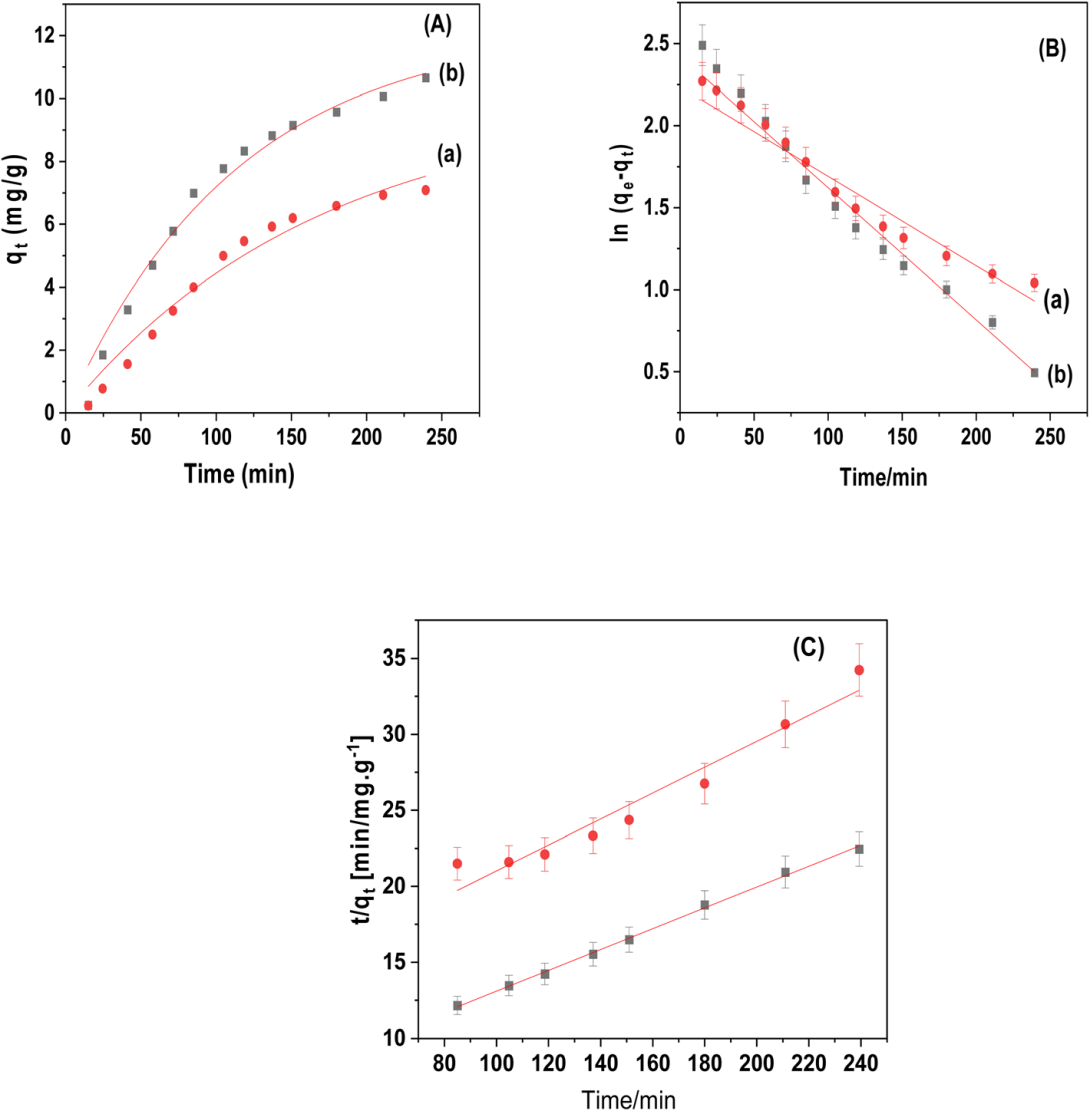
(A) Kinetic models of IMD adsorption onto CuFe_2_O_4_ and CuFe_2_O_4_/Am-rGO composite; (B) kinetics plot of pseudo first order; and (C) kinetics plot of pseudo second order [conditions: initial conc. of IMD solution = 5.0 mg L^−1^, adsorbent dose = 0.02 g, pH = 5.3, volume = 50 mL].

Fig. 9a–c and Table 1 provided the non-linear kinetic fitting curves and all parameters of the pseudo-first order and pseudo-second order models, respectively. Compared to the pseudo-first-order models, the pseudo-second-order model for CuFe_2_O_4_ and CuFe_2_O_4_/Am-rGO composite showed larger non-linear correlation coefficients (*R*^2^ = 0.968–0.997), which allowed it to more accurately reflect the kinetic data. In agreement with the experimental data (*q*_e,exp_), the equilibrium adsorption quantity (*q*_e,cal_) computed from the pseudo-second-order model also supported this. This finding implied that IMD adsorption involved chemisorption (valence force, π–π conjugation, and hydrogen bonding).^38,39^

**Table 1:** Adsorption kinetics parameters using pseudo first and second order models

Adsorbent	Pseudo-first order			Pseudo-second order		
	*k*_1_ (min^−1^)	*q*_e1_ (mg g^−1^)	*R* ^2^	*k*_2_ [g (mg min)^−1^]	*q*_e2_ (mg g^−1^)	*R* ^2^
CuFe_2_O_4_	5.94 × 10^−3^	9.92	0.939	5.6 × 10^−4^	11.72	0.968
CuFe_2_O_4_/Am-rGO	8.79 × 10^−3^	12.3	0.978	7.5 × 10^−4^	14.57	0.997

The equation *h* = *k*_2_*q*_e2_ can be used to get the initial adsorption rate [*h* (μg g^−1^ min^−1^)] of the pseudo-second-order model. It was clear that CuFe_2_O_4_/Am-rGO had a higher *h* value than CuFe_2_O_4_. This means that hydrogen bonding or π–π electron donor–acceptor interaction helped move the mass between CuFe_2_O_4_/Am-rGO and the IMD solution.^38^

The initial rate for pseudo-first order can be determined as the product of the rate constant *k*_1_ and the initial adsorbate concentration. It was calculated to be 2.97 × 10^−2^ and 4.39 × 10^−2^ mg g^−1^ min^−1^ for CuFe_2_O_4_ and CuFe_2_O_4_/Am-rGO, respectively. The initial adsorption rate (*r*_0_) can be calculated using the following equation derived from the pseudo-second-order model:*r*_0_ = *k*_2_ × *q*_e2_^2^

It was calculated to be 7.69 × 10^−2^ and 0.159 mg g^−1^ min^−1^ for CuFe_2_O_4_ and CuFe_2_O_4_/Am-rGO, respectively.

### Adsorption isotherms

**Fig. 10:**
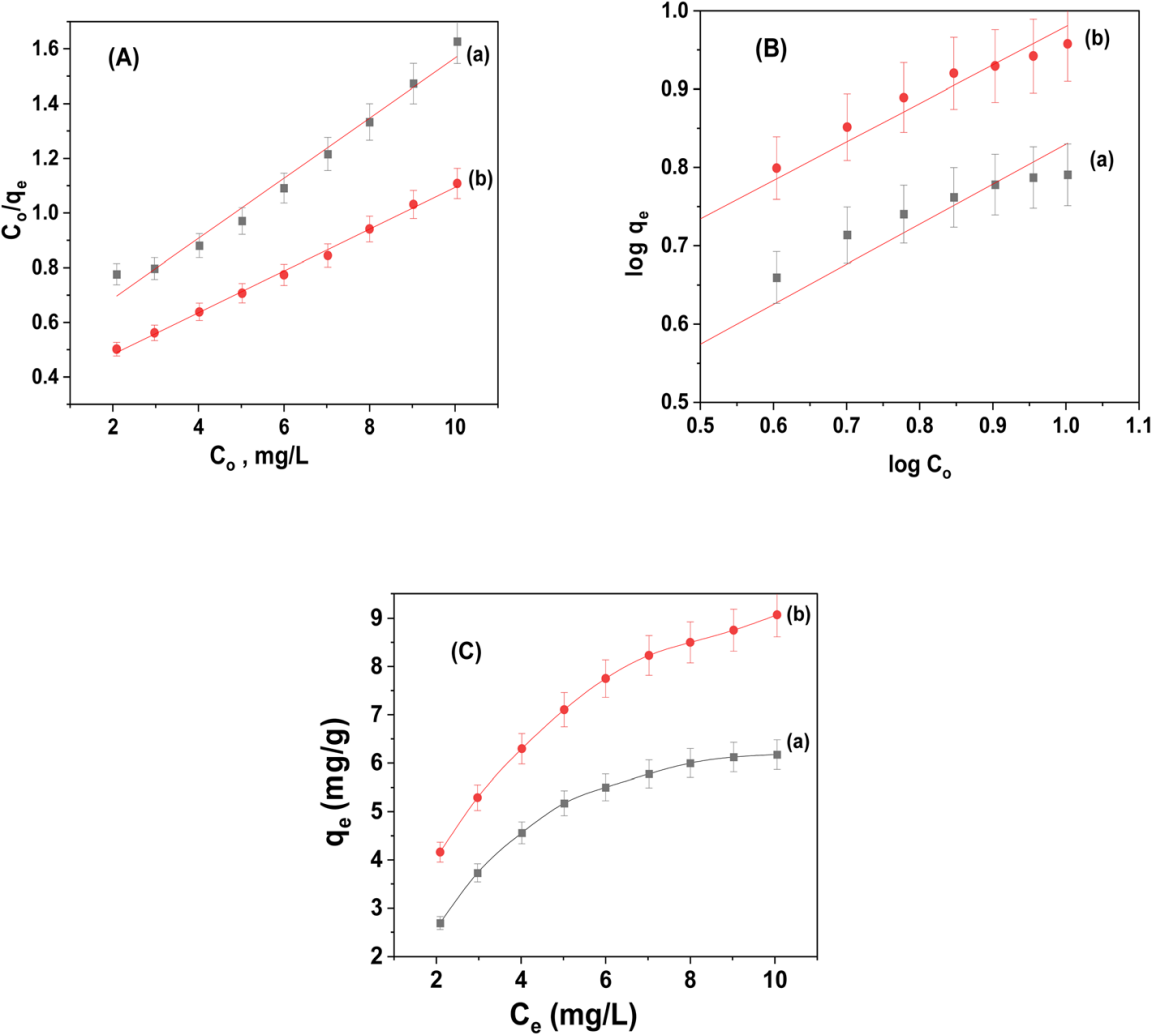
Adsorption isotherm for the adsorption of IMD onto (a) CuFe_2_O_4_ and (b) CuFe_2_O_4_/Am-rGO composites: (A) Langmuir, (B) Freundlich models and (C) *C*_e_*vs. Q*_e_ plot.

As illustrated in Fig. 10, the realistic adsorption isotherm data was identified using the Langmuir and Freundlich isotherms. Table 2 presents an illustration of the isotherm parameters. The correlation coefficients (*R*^2^) of the linear plot revealed that both adsorbents obeyed Langmuir isotherms, confirming the existence of homogenous adsorption processes and the creation of monolayers on the surface. The maximal capacity per unit mass (*Q*_m_) for CuFe_2_O_4_ and CuFe_2_O_4_/Am-rGO composites, according to the Langmuir model, was 9.095 ± 0.6 and 13.11 ± 1.5 mg g^−1^, respectively.

**Table 2:** Adsorption isotherm parameters using Langmuir and Freundlich models

Adsorbent	Langmuir model			Freundlich model		
	*Q*_m_ (mg g^−1^)	*b* (mg^−1^)	*R* ^2^ (*n* = 5)	*K*_F_ (mg^(*n*−1)/*n*^ L^1/*n*^ g^−1^)	*n*	*R* ^2^ (*n* = 5)
CuFe_2_O_4_	9.09	0.24	0.977	2.08	1.95	0.923
CuFe_2_O_4_/Am-rGO	13.1	0.23	0.996	3.07	2.03	0.971

### Reusability

**Fig. 11:**
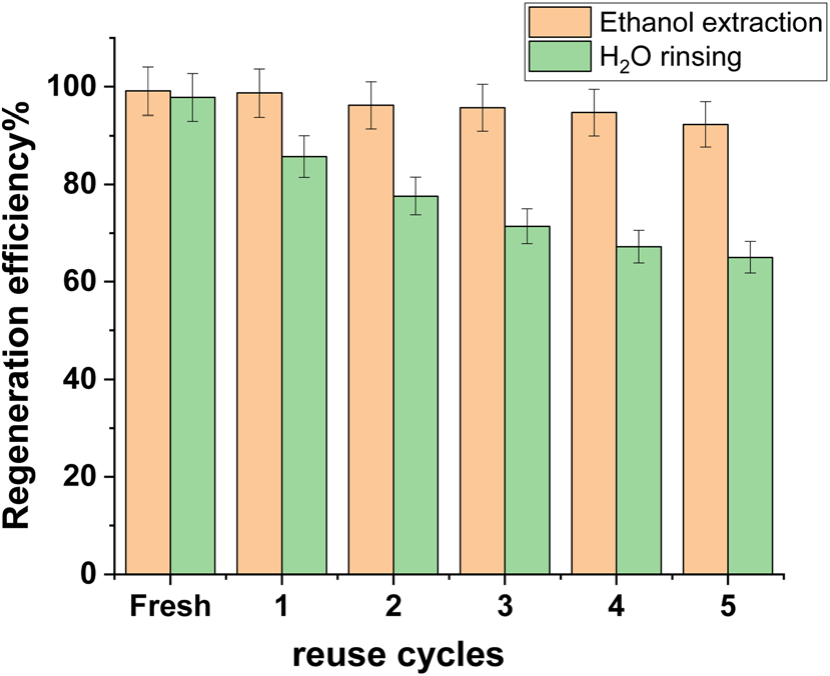
Ethanol extraction and H_2_O rinse result in a regeneration efficiency of CuFe_2_O_4_/Am-rGO.

Regeneration of adsorbents provided viability for their sustainable adsorption.^38^ The extraction of organic solvents (methanol, ethanol, and acetone) was thought to be a good way to regenerate the adsorbent that was used.^40^ Because of its good extraction performance and low toxicity, ethanol was employed in this investigation to regenerate the CuFe_2_O_4_/Am-rGO composite, with H_2_O-rinsing serving as the control. It was clear that the CuFe_2_O_4_/Am-rGO regeneration efficiency with H_2_O-rinsing decreased with the number of reuse cycles; after five cycles, it was only 64.83% of what it was when it was first made (Fig. 11). At the same time, ethanol extraction effectively preserved CuFe_2_O_4_/Am-rGO's stable, durable adsorption capability (>90% of the fresh one). If you extracted CuFe_2_O_4_/Am-rGO with ethanol, it was better at regeneration than when you extracted granular activated carbon with ethanol (60% of its initial adsorption capacity for 2,4-dinitrophenol)^41^ or pine-wood biochar with methanol (76% and 72% of its initial adsorption capacity for salicylic acid and ibuprofen, respectively).^42^

### Mechanism of adsorption

**Fig. 12:**
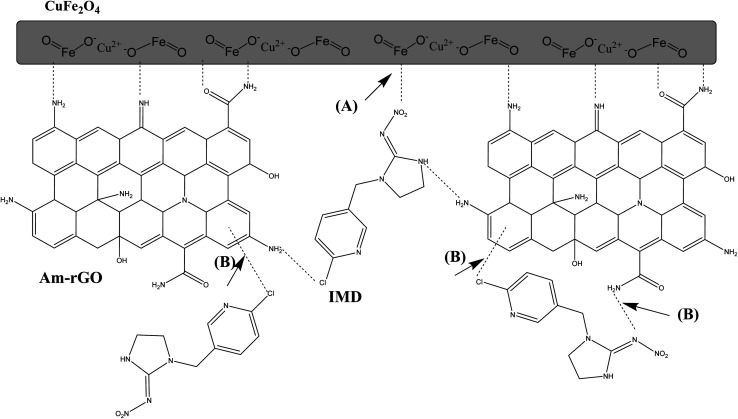
Schematic of IMD adsorption mechanism on CuFe_2_O_4_/Am-rGO composite, (A) a physical adsorption that occurs in the adsorbent's porosity or on the surface of the Am-rGO layer, and (B) adsorption *via* interactions between the IMD and the Am-rGO layer.

The process by which the CuFe_2_O_4_/Am-rGO composite gets rid of IMD is shown in Fig. 12. Here, two distinct hypothesized pathways for the adsorption of IMD on the CuFe_2_O_4_/Am-rGO composite surface are shown. The first is a physical adsorption that occurs in the adsorbent's porosity or on the surface of the Am-rGO layer. The last pathway involves adsorption *via* interactions between the IMD and the Am-rGO layer. Graphite carbon served as the giver of π electrons, while the hetero ring of IMD, comprising nitrogen and chlorine, served as the acceptor of π electrons, promoting the π–π conjugation.^27,43^ Nanoparticles of CuFe_2_O_4_ exhibit a large specific surface area. The adsorbent's pores let IMD pass through and stick to the CuFe_2_O_4_ nanoparticle surface.

### Evaluation of imidacloprid's adsorption capacity relating to other adsorbents

In this work, the adsorbents utilized for the adsorption of imidacloprid pesticide were compared to those that had been previously reported, as shown in Table 3. The functional group and type of precursor employed are two factors that affect how different adsorbents have variable adsorption capacities. In this work, CuFe_2_O_4_/Am-rGO has proven to be an effective adsorbent for removing imidacloprid from contaminated water.

**Table 3:** A Comparative study of IMD adsorption using different adsorbents^a^^b^^c^^d^^e^

Adsorbents	Batch experiment conditions			pH	Adsorption capacity (mg g^−1^)	Model fitting	IMD sorption mechanism	Ref.
	Co (mg L^−1^)	*t* (h)	*m* (mg)					
Agri-waste derived biochar	1–10	24 h	1.0	—	—	PFO, PSO, F, ID	Pore filling, electrostatic interaction, hydrophobic interaction	15
Silver@graphene oxide	10	1	0.6	6.6	25.7	F, PSO	Electrostatic and hydrophobic interaction	36
Activated carbon	10	—	—	5.2	7.78–39.4	L, F, PFO, PSO, ID	Hydrogen bonding and π–π interaction	44
Biochar	20	6	2	—	18.2–23.8	PFO, PSO, L, F	Ionic and hydrophobic interaction	19
Biochar	20	12	0.1	—	8–15	L, F, PFO, PSO	Pore filling, π–π stacking, and polar interaction	
GO/CoFe_2_O_4_-SBC	0.1	2 h	0.01	6.0	8.64	F, T, PSO	Pore filling, π–π conjugation and functional groups interaction	35
Fe/Zn + KOH/BC	20	12	0.1	—	160–185	L, F, PFO, PSO	Pore filling, π–π and polar interaction	45
Iron and base modified biochar	23.8	21	0.5–10	—	10.6	L, F, PSO	Pore filling, polar interaction, and π–π and interaction	46
KOH/biochar	20	12	0.1	—	60–67	L, F, PFO, PSO	Pore filling, π–π stacking, and polar interaction	45
KOH + Fe/Zn-LBC	20	12	0.05	5	738	L	Pore filling, hydrogen bonding and π–π conjugation	47
Eucalyptus wood chips biochar	500	—	—	7	14.7	PSO, F	π-π conjugation and functional groups interaction	48
HAC	10–100	2	0.1	1–8	76.9	PSO, F, L, T	—	49
WAC	10–100	2	0.1	1–8	83.3	PSO, F, L, T	—	
CuFe_2_O_4_	0.5	2	0.02	5.3	9.09	PSO, L, F	Pore filling and functional groups interaction	This work
CuFe_2_O_4_/Am-rGO	0.5	2	0.02	5.3	13.1		Pore filling, π–π conjugation and functional groups interaction	

a GO/CoFe_2_O_4_-SBC: graphene oxide supported magnetic sludge biochar composite.

b KOH + Fe/Zn-LBC: potassium hydroxide activated magnetic microporous loofah sponge biochar.

c HAC: nut shells of hazelnut.

d WAC: walnut.

e C0, initial concentration; *t*, contact time; *d*, adsorbent dose; L, Langmuir; F, Freundlich; PFO, pseudo-first order; PSO, pseudo-second order; and ID, intra-particle diffusion.

## Conclusions

In our research, we designed the new material CuFe_2_O_4_/Am-rGO nanocomposite to remediate IMD pesticide for the first time. This material has all the advantages of CuFe_2_O_4_ and Am-rGO. It exhibits excellent adsorption properties for IMD from aqueous solutions due to the more suspended hydroxyl, carboxylic, and amino groups on its surface (*i.e.*, a large adsorption capacity and a quick adsorption rate). These properties can create an intense interaction with IMD through chemical effects (valence force, π–π conjugation, and hydrogen bonding). After conducting XRD, EDX, SEM, and BET studies, it was determined that the CuFe_2_O_4_/Am-rGO nanocomposite had been successfully synthesized. It has been discovered that the adsorption capability in batch mode depends on the adsorbent dose, solution pH, equilibration period, and initial IMD concentration. The maximum amount of adsorption capacity is close to 13.11 ± 1.5 mg g^−1^. Adsorption isotherm tests show that the CuFe_2_O_4_/Am-rGO nanocomposite can quickly remove IMD from water solutions, with a rate of more than 97%. Therefore, CuFe_2_O_4_/Am-rGO nanocomposite materials are excellent choices for IMD pollution removal in natural settings. In conclusion, the alteration of ferrites using the Am-rGO nanocomposite may, soon, bring about a revolution in the treatment of water pollution.

## Data availability

Data will be made available on request.

## Author contributions

Ayman H. Kamel: conceptualization, supervision, data curation, formal analysis, visualization, methodology, data curation, validation, writing – original draft. Hisham S. M. Abd-Rabboh: methodology, data curation, funding acquisition, project administration, supervision, writing – review & editing, resource and investigation.

## Conflicts of interest

The authors declare that they have no known competing financial interests or personal relationships that could have appeared to influence the work reported in this paper.
